# 紫杉醇脂质体诱导加同期放化疗与序贯放化疗治疗局部晚期非小细胞肺癌的随机对照研究

**DOI:** 10.3779/j.issn.1009-3419.2011.02.06

**Published:** 2011-02-20

**Authors:** 幼艺 戴, 武忠 姜, 君 袁, 瑞 魏

**Affiliations:** 410008 长沙，中南大学湘雅医院肿瘤科 Department of Oncology, Xiangya Hospital, Central South University, Changsha 410008, China

**Keywords:** 肺肿瘤, 紫杉醇, 放疗, 化疗, 综合治疗, Lung neoplasms, Paclitaxel, Radiotherapy, Chemotherapy, Combined modality therapy

## Abstract

**背景与目的:**

序贯放化疗及同期放化疗在局部晚期肺癌治疗中得以广泛研究，而诱导加同期放化疗的研究尚少。紫杉醇脂质体副作用少，可使诱导加同期放化疗更顺利地实施。本文旨在比较紫杉醇脂质体加顺铂（TP方案）诱导加同期放化疗和序贯放化疗治疗局部晚期非小细胞肺癌（non-small cell lung cancer, NSCLC）的疗效及毒副作用。

**方法:**

我院60例局部晚期NSCLC患者随机分为诱导加同期放化疗组（A组）和序贯放化疗组（B组）。A组：诱导化疗2个-3个周期后行同期放化疗，放疗的第1天及第22天予TP方案化疗（紫杉醇脂质体135 mg/m^2^-175 mg/m^2^，d1；顺铂70 mg/m^2^-80 mg/m^2^，d2），期间持续放疗。B组：化疗方案同前，化疗4个-6个周期后，行放疗。两组放疗方式均为三维适形放疗，总剂量为56 Gy-70 Gy。观察和比较两组的疗效和毒副作用。

**结果:**

A组、B组总有效率分别为80.3%和60%，组间有统计学差异（*P*=0.042）；1年生存率分别为71.4%和53.2%，组间无统计学差异（*P*=0.18）；骨髓抑制发生率分别为90%和73.3%，组间无统计学差异（*P*=0.09）；放射性食管炎发生率分别为50%和36.7%，组间无统计学差异（*P*=0.147）；肺纤维化发生率分别为30%和20%，组间无统计学差异（*P*=0.276）。

**结论:**

在晚期NSCLC的局部治疗中，TP方案诱导加同期放化疗较序贯放化疗的近期疗效好，但毒副反应无明显区别。

## Abstract (en)

**Background and objective:**

Sequential and concurrent chemoradiotherapy were widely studied in locally advanced non-small cell lung cancer (NSCLC), but the reports of induction chemotherapy followed concurrent chemoradiotherapy are rare so far. The little side effects of paclitaxel liposme may be convenient to carry out induction chemotherapy followed concurrent chemoradiotherapy. The aim of this study is to compare the effects and side effects of TP regimen (Paclitaxel liposme and cisplatin) induction chemotherapy followed concurrent chemoradiotherapy with sequential radiotherapy on locally advanced NSCLC.

**Methods:**

Sixty locally advanced NSCLC patients were randomly divided into group A, induction chemotherapy followed concurrent chemoradiotherapy and group B, sequential radiotherapy group. The patients in group A received 2-3 cycles of induced chemotherapy included of Paclitaxel liposme 135 mg/m^2^-175 mg/m^2^, d1 and cisplatin 70 mg/m^2^-80 mg/m^2^, d2, 3 weeks repeat and after 2-3 cycles followed by concurrent chemoradiotherapy. The patients in group B received chemotherapy, (as described above in group A) 4-6 cycles of chemotherapy followed one cycle of radiotherapy. The three-dimensional conformal radiotherapy at the total dose of 56 Gy-70 Gy was applied in all patients.

**Results:**

The response rate in group A and group B were 80.3% and 60%, respectively (*P*=0.042). 1-year survival rates were 71.4% and 53.2%, respectively (*P*=0.18). And there were no significant difference of myelosuppression, radiation esophagitis and pulmonary fibrosis between the two groups (*P*=0.09, *P*=0.147, *P*=0.276, respectively).

**Conclusion:**

The recent effects of induction chemotherapy followed by concurrent chemoradiotherapy group were better than sequential radiotherapy group on locally advanced NSCLC and there was no significant difference in side effects between the two groups.

序贯放化疗及同期放化疗在局部晚期肺癌治疗中得以广泛研究，而有关诱导加同期放化疗的研究尚少。紫杉醇脂质体联合顺铂(TP方案)治疗晚期非小细胞肺癌(non-small cell lung cancer, NSCLC)近期疗效确切，毒副反应比传统紫杉醇联合顺铂轻^[1]^，而TP方案诱导加同期放化疗是否既可以顺利实施，又能提高疗效？为此，本研究前瞻性比较分析我院60例局部晚期NSCLC患者采用TP方案诱导加同期放化疗和序贯放化疗的疗效及毒副作用，以期指导临床实践。

## 1 材料与方法

### 1.1 病历资料

收集2007年3月-2009年9月我院收治的60例经病理证实为NSCLC的患者，进行全身检查后根据世界卫生组织(World Health Organization, WHO)1997 TNM分期为Ⅲa/Ⅲb期，失去手术机会或拒绝手术，其中鳞癌36例，腺癌18例，腺鳞癌4例，类癌2例，年龄在34岁-72岁，中位年龄为56岁，既往无化疗及胸部放疗史，*Karnofsky*体能状态评分 > 70分。其中1例合并频发室性早搏，4例合并2型糖尿病。采用电脑随机数生成法将患者随机分为TP方案诱导加同期放化疗组(A组)30例，序贯治疗组(B组)30例，分组后年龄、性别、分期情况经统计分析具有可比性。

### 1.2 治疗方法

A组：先行TP方案(紫杉醇脂质体135 mg/m^2^-175 mg/m^2^，d1，顺铂70 mg/m^2^-80 mg/m^2^，d2，3周重复)诱导化疗2个-3个周期后，行同期放化疗，放疗的第1天及第22天予TP方案化疗(用药同前)，放疗不间断。序贯放化疗(B组)：TP化疗方案同A组，共化疗4个-6个周期后，行三维适形放疗(three-dimensional conformal radiotherapy, 3D CRT)一疗程。

放疗：采用全程CRT，设备为Varian eclipse 2000直线加速器，射线能量为6 MV X线，患者取仰卧位，双手抱头，CT定位，Dicom数据传输系统进行数据传输，真空袋体位固定，CT模拟和三维治疗计划系统(three-dimensional treatment planning system, 3D TPS)设计放疗计划。靶区按照ICRU 62号文件规定定义：大体肿瘤体积(gross tumor volume, GTV)范围包括原发灶、阳性淋巴结，以化疗后影像为依据设定CTV与GTV，而且CTV包全化疗前瘤床(CT扫描短径≥1 cm或PET阳性)；临床靶体积(clinical target volume, CTV)是鳞癌或腺癌GTV边缘分别外扩6 mm及8 mm，其它病理类型者GTV外扩8 mm；不做淋巴结区预防性照射。计划靶体积(planning target volume, PTV)为CTV外扩1.0 cm-1.5 cm。90%-95%等剂量曲线包绕90%-95%的PTV，常规分割，每天1次，每周5次，每次2 Gy，共面设计4个-7个照射野，尽量避开脊髓和正常肺组织，非均匀分布，肺V20(接受≥20 Gy照射剂量的肺体积占总肺体积的百分比)≤30%，脊髓受量≤45 Gy的条件下尽可能提高，心脏V_40_ < 40%。PTV总剂量在56 Gy-70 Gy。

### 1.3 疗效观察及毒副作用评价

依据常用药物毒性标准V3.0和RTOG放射性损伤分级标准进行毒副作用评价。同期放化疗过程中每周复查血常规，治疗前后做心电图，治疗过程中密切观察食管反应(3例食管反应明显的进行食管镜检查)，放疗结束时及放疗后每3个月定期复查肺部CT和心电图。肺纤维化依据复查胸片及肺部CT出现节段性的肺纹理增多紊乱，条索状影，肺组织萎缩，有的可伴有肺大泡。疗效评价标准按照WHO实体瘤疗效评价标准分为完全缓解(complete response, CR)、部分缓解(partial response, PR)、无变化(no change, NC)和进展(progressive disease, PD)。CR为病灶完全消失，PR为病灶缩小50%以上，NC为病灶病灶缩小不到50%或增大不超过25%；增大超过25%以上为PD。

60例患者治疗后第1年每3个月随访1次，第2年以上每半年随访1次。截止2010年3月1日，中位随访时间12个月-32个月，无1例失访，随访率为100%。

### 1.4 统计方法

生存期从接受3D CRT和同期化疗算起，采用SPSS 13.0统计软件行*Kaplan-Meier*法计算生存率。两组之间率的比较采用*Fisher*确切概率法。以*P* < 0.05为有统计学差异。

## 2 结果

### 2.1 近期疗效比较

A组、B组总有效率(CR+PR)分别为80.3%和60%，组间有统计学差异(*P*=0.042)。A组、B组无效率(NC+PD)分别为16.7%和40%，组间有统计学差异(*P*=0.042)(表 1)。

**1:** 诱导加同期放化疗组（A组）与序贯放化疗组（B组）近期疗效的比较 Comparison of the recent effects between induction chemotherapy followed concurrent chemoradiotherapy and sequential radiotherapy groups

Group	*n*	CR	PR	NC	PD
A	30	3 (10.0%)	22 (70.3%)	3 (10.0%)	2 (6.7%)
B	30	1 (3.3%)	17 (56.7%)	8 (26.7%)	4 (13.3%)
The total effects between the two groups analyzed by *Fisher* exact probability test, *P*=0.042. CR: complete response; PR: partial response; NC: no change; PD: progressive disease; A: indicate induction chemotherapy followed concurrent chemoradiotherapy group; B: indicate sequential radiotherapy group.					

### 2.2 远期疗效比较

A组、B组1年生存率分别为71.4%和53.2%，组间无统计学差异(*P*=0.18)(图 1)。

**1:**
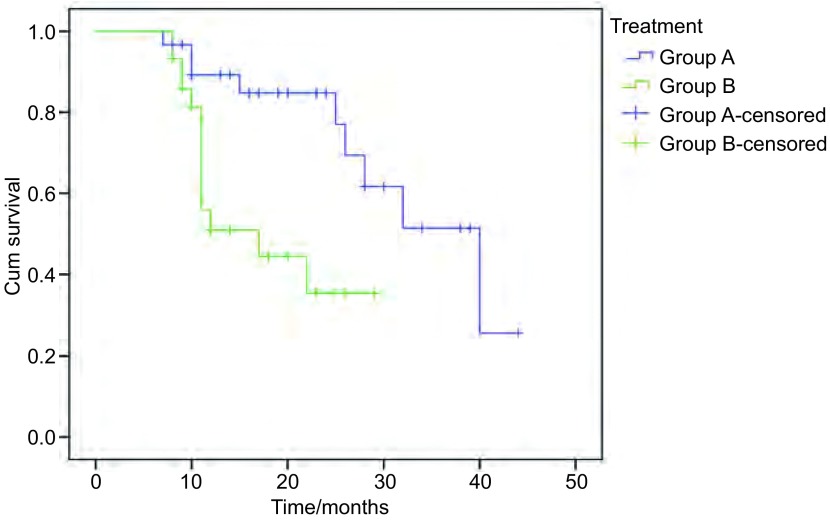
诱导加同期放化疗组（A组）与序贯放化疗组（B组）的生存曲线 Survival curve of induction chemotherapy followed concurrent chemoradiotherapy and sequential radiotherapy

### 2.3 不良反应

A组、B组骨髓抑制发生率分别为90%和73.3%，组间无统计学差异(*P*=0.09)；放射性食管炎发生率分别为50%和36.7%，组间无统计学意义(*P*=0.147)。肺纤维化发生率分别为30%和20%，组间无统计学差异(*P*=0.276)。15例肺纤维化患者，表现为照射野区肺纹理增多增粗，其中10例无临床症状，仅5例患者出现了急性放射性肺炎(3例1级，1例2级，1例3级)表现，经抗炎治疗(未用激素治疗)后感染控制。全组病例未出现心血管不良事件。所有病例未发生严重不良副反应(表 2)。

**2:** 诱导加同期放化疗组（A组）与序贯放化疗组（B组）不良反应的比较 Comparison of side effects between induction chemotherapy followed concurrent chemoradiotherapy and sequential radiotherapy groups

Group	Bone marrow suppression				Radiation esophagitis	Pulmonary fibrosis
	Ⅰ	Ⅱ	Ⅲ	Ⅳ		
A	18	9	0	0	15 (50.0%)	9 (30.0%)
B	15	6	1	0	11 (36.7%)	6 (20.0%)
The *P* value between two groups were 0.09, 0.147 and 0.276, respectively, in compared bone marrow suppression, radiation esophagitis and pulmonary fibrosis.						

## 3 讨论

参考文献NSCLC占肺癌总数的75%-80%，由于缺乏早期诊断方法，因此确诊时75%的患者属于中晚期，失去手术机会^[2]^。化疗加放疗的综合治疗可能提高疗效，延长患者的生存期^[3]^。对于局部晚期NSCLC患者进行序贯放化疗还是同期放化疗一直是最近的研究热点。本组研究将诱导化疗与同期放化疗结合，其原因是局部晚期NSCLC肿块巨大，先诱导化疗2个-3个周期使肿瘤缩小，可以缩小放疗时的照射体积，而后的同期放化疗在强化局部治疗的同时兼顾到全身微小转移灶的治疗，放化疗相互协同，化疗药物能提高肿瘤细胞对放疗的敏感性，放疗也可增强化疗药物的细胞毒性，从而增强了对局部肿瘤的杀伤作用，避免了肿瘤细胞在放疗后的加速再增殖。与序贯放化疗进行相比，同步放化疗使总疗程缩短，从而提高生活质量，节省治疗费用^[4]^。1999年西日本肺癌组比较同期放化疗和序贯放化疗的生存，结果表明同期放化疗具有优势^[5]^。常用的同期放化疗方案有每周方案和三周方案。同期放化疗中，化疗药物在增加放疗效应的同时也增加正常组织的损伤，导致副作用增加。患者能否耐受治疗是治疗方案选择时首先要考虑的重要因素。有研究^[6]^发现诱导化疗后，3D CRT结合每周紫杉醇治疗局部晚期NSCLC能为大多数患者耐受，初步疗效令人鼓舞。同期化疗药物紫杉醇既有抗癌活性，又有放疗增敏的作用。其增敏机制可能在于使肿瘤细胞同步于G/M_2_期，有利于射线杀灭肿瘤细胞^[7, 8]^，同时促进了细胞凋亡的发生^[9]^。本研究把诱导化疗与同期放化疗相结合，旨在结合两者的优势，进一步提高肺癌的疗效，研究发现1年生存率与序贯放化疗无异，但总有效率优于序贯放化疗。

脂质体紫杉醇是以脂质体作为药物载体，将紫杉醇包埋入其中，可溶于水，克服了紫杉醇不溶于水引起的问题。脂质体紫杉醇为主化疗治疗晚期NSCLC疗效与普通紫杉醇相当，因在治疗过程中避免了有机溶剂与大剂量激素的使用，减少了与此相关的不良反应^[10]^，为同期放化疗的顺利实施提供了可能。本组研究选用药物为紫杉醇脂质体加顺铂联合的TP方案联合放疗，无论是诱导加同期放化疗组还是序贯放化疗组，全部患者(包括1例合并频发室性早搏，4例合并2型糖尿)均未发生严重毒副作用，顺利完成治疗。

3D CRT即高剂量区分布形状在三维方向上与病变(靶区)的形状一致，正常组织受量明显减少。肺癌的常规放疗一般采用两野对穿照射，基于正常肺组织及脊髓的剂量限值，局部肿瘤剂量难以提高。Kong等^[11]^研究高放射剂量是否可以改善局部晚期NSCLC局控率和生存率问题，结果显示放射剂量是肿瘤局部控制和患者生存率决定因素，每增加1 Gy剂量，患者3年-5年局部控制率提高1%。而3D CRT则采用多个照射野，这样在保证肿瘤受量的同时最大限度地降低了正常组织受量，为提高肿瘤局部的放射剂量奠定了基础，利用适形放疗技术最大限度地提高了肿瘤区的照射剂量。

本组研究采用TP方案结合三维适形对局部晚期NSCLC行诱导加同期放化疗，与序贯放化疗相比，并未增加毒副反应。诱导加同期放化疗组总有效率优于序贯放化疗组，但1年生存率两组间无统计学差异，但是前者明显提高了治疗有效率，而且总治疗时间有所缩短。总之，紫杉醇脂质体结合三维适形放疗诱导加同期治疗局部晚期NSCLC提高了治疗有效率，其副作用可以接受。
